# Supplementary material for: Maternal exposure to SSRIs or SNRIs and the risk of congenital abnormalities in offspring: A systematic review and meta-analysis
Source: PLoS One. 2023 Nov 29;18(11):e0294996. doi: 10.1371/journal.pone.0294996 (PMC10686472; doi:10.1371/journal.pone.0294996)
Supplement: S1 Appendix — (DOCX) [file pone.0294996.s001.docx]

S1 Appendix. Literature search strategy

Search strategy for PubMed (January 2000 to May 2023)

1. Abnormality, Congenital OR Congenital Abnormality OR Deformities OR Deformity OR Congenital Defects OR Congenital Defect OR Defect, Congenital OR Defects, Congenital OR Abnormalities, Congenital OR Birth Defects OR Birth Defect OR Defect, Birth OR Fetal Malformations OR Fetal Malformation OR Malformation, Fetal OR Fetal Anomalies OR Anomaly, Fetal OR Fetal Anomaly (772,138)

2. Fluoxetin OR N-Methyl-gamma-(4-(trifluoromethyl)phenoxy)benzenepropanamine OR Lilly-110140 OR Lilly 110140 OR Lilly110140 OR Sarafem OR Fluoxetine Hydrochloride OR Prozac (11,266)

3. Sertraline OR Zoloft OR Lustral OR Altruline OR Apo-Sertraline OR Apo Sertraline OR Aremis OR Besitran OR Sealdin OR Gladem OR Novo-Sertraline OR Novo Sertraline OR ratio-Sertraline OR ratio Sertraline OR Rhoxal-sertraline OR Rhoxal sertraline OR Sertraline Hydrochloride OR Hydrochloride, Sertraline OR Sertraline Hydrochloride (1S-cis)-Isomer OR Gen-Sertraline OR Gen Sertraline (5,018)

4. Cytalopram OR Citalopram Hydrobromide OR Lu-10-171 OR Lu10171 OR Seropram OR Celexa (7,703)

5. Selective Serotonin Reuptake Inhibitor OR SSRIs OR Serotonin Uptake Inhibitors OR Reuptake Inhibitors, Serotonin OR Uptake Inhibitors, 5-Hydroxytryptamine OR Uptake Inhibitors, Serotonin OR Serotonin Reuptake Inhibitor OR Inhibitor, Serotonin Reuptake OR Reuptake Inhibitor, Serotonin OR Serotonin Uptake Inhibitor OR Inhibitor, Serotonin Uptake OR Uptake Inhibitor, Serotonin OR 5-HT Uptake Inhibitor OR Inhibitor, 5-HT Uptake OR Uptake Inhibitor, 5-HT OR 5-Hydroxytryptamine Uptake Inhibitor OR Inhibitor, 5-Hydroxytryptamine Uptake OR Uptake Inhibitor, 5-Hydroxytryptamine OR 5-HT Uptake Inhibitors OR 5-Hydroxytryptamine Uptake Inhibitors OR Inhibitors, 5-HT Uptake OR Inhibitors, 5-Hydroxytryptamine Uptake OR Inhibitors, Serotonin Reuptake OR Inhibitors, Serotonin Uptake OR Serotonin Reuptake Inhibitors OR Uptake Inhibitors, 5-HT (41,676 )

6. Serotonin and Norepinephrine Reuptake Inhibitors OR SSRIs and NRIs OR NRIs and SSRIs OR Serotonin and Noradrenaline Uptake Inhibitors OR SNRIs OR SNRI OR Serotonin and Norepinephrine Uptake Inhibitors (8,613)

7. Hydrochloride, Venlafaxine OR Cyclohexanol, 1-(2-(dimethylamino)-1-(4-methoxyphenyl)ethyl)-, hydrochloride OR 1-(2-(dimethylamino)-1-(4-methoxyphenyl)ethyl)cyclohexanol HCl OR Wy 45030 OR Wy-45030 OR Wy45030 OR Wy-45,030 OR Wy 45,030 OR Wy45,030 OR Sila-Venlafaxine OR Sila Venlafaxine OR Effexor OR Trevilor OR Vandral OR Efexor OR Venlafaxine OR Dobupal (4,546)

8. Hydrochloride, Duloxetine OR Duloxetine HCl OR HCl, Duloxetine OR LY 248686 OR LY-248686 OR LY248686 OR Duloxetine Ethanedioate (1:1), (+-)-isomer - T353987 OR LY 227942 OR LY-227942 OR LY227942 OR Duloxetine OR N-methyl-3-(1-naphthalenyloxy)-3-(2-thiophene)propenamide OR N-methyl-3-(1-naphthalenyloxy)-2-thiophenepropanamine Duloxetine, (+)-isomer OR Cymbalta (3,236)

9. (#1 AND #2) OR (#1 AND #3) OR (#1 AND #4) OR (#1 AND #5) OR (#1 AND #6) OR (#1 AND #7) OR (#1 AND #8) (1,691)

Search strategy for Web of Science (January 2000 to May 2023)

1. TS= (Abnormality, Congenital OR Congenital Abnormality OR Deformities OR Deformity OR Congenital Defects OR Congenital Defect OR Defect, Congenital OR Defects, Congenital OR Abnormalities, Congenital OR Birth Defects OR Birth Defect OR Defect, Birth OR Fetal Malformations OR Fetal Malformation OR Malformation, Fetal OR Fetal Anomalies OR Anomaly, Fetal OR Fetal Anomaly) (154,077)

2. TS= (Fluoxetin OR N-Methyl-gamma-(4-(trifluoromethyl)phenoxy)benzenepropanamine OR Lilly-110140 OR Lilly 110140 OR Lilly110140 OR Sarafem OR Fluoxetine Hydrochloride OR Prozac) (1,136)

3. TS= (Sertraline OR Zoloft OR Lustral OR Altruline OR Apo-Sertraline OR Apo Sertraline OR Aremis OR Besitran OR Sealdin OR Gladem OR Novo-Sertraline OR Novo Sertraline OR ratio-Sertraline OR ratio Sertraline OR Rhoxal-sertraline OR Rhoxal sertraline OR Sertraline Hydrochloride OR Hydrochloride, Sertraline OR Sertraline Hydrochloride (1S-cis)-Isomer OR Gen-Sertraline OR Gen Sertraline) (6,840)

4. TS= (Cytalopram OR Citalopram Hydrobromide OR Lu-10-171 OR Lu10171 OR Seropram OR Celexa) (120)

5. TS= (Selective Serotonin Reuptake Inhibitor OR SSRIs OR Serotonin Uptake Inhibitors OR Reuptake Inhibitors, Serotonin OR Uptake Inhibitors, 5-Hydroxytryptamine OR Uptake Inhibitors, Serotonin OR Serotonin Reuptake Inhibitor OR Inhibitor, Serotonin Reuptake OR Reuptake Inhibitor, Serotonin OR Serotonin Uptake Inhibitor OR Inhibitor, Serotonin Uptake OR Uptake Inhibitor, Serotonin OR 5-HT Uptake Inhibitor OR Inhibitor, 5-HT Uptake OR Uptake Inhibitor, 5-HT OR 5-Hydroxytryptamine Uptake Inhibitor OR Inhibitor, 5-Hydroxytryptamine Uptake OR Uptake Inhibitor, 5-Hydroxytryptamine OR 5-HT Uptake Inhibitors OR 5-Hydroxytryptamine Uptake Inhibitors OR Inhibitors, 5-HT Uptake OR Inhibitors, 5-Hydroxytryptamine Uptake OR Inhibitors, Serotonin Reuptake OR Inhibitors, Serotonin Uptake OR Serotonin Reuptake Inhibitors OR Uptake Inhibitors, 5-HT) (25,483)

6. TS = (Serotonin and Norepinephrine Reuptake Inhibitors OR SSRIs and NRIs OR NRIs and SSRIs OR Serotonin and Noradrenaline Uptake Inhibitors OR SNRIs OR SNRI OR Serotonin and Norepinephrine Uptake Inhibitors) (4,591)

7. TS= (Hydrochloride, Venlafaxine OR Cyclohexanol, 1-(2-(dimethylamino)-1-(4-methoxyphenyl)ethyl)-, hydrochloride OR 1-(2-(dimethylamino)-1-(4-methoxyphenyl)ethyl)cyclohexanol HCl OR Wy 45030 OR Wy-45030 OR Wy45030 OR Wy-45,030 OR Wy 45,030 OR Wy45,030 OR Sila-Venlafaxine OR Sila Venlafaxine OR Effexor OR Trevilor OR Vandral OR Efexor OR Venlafaxine OR Dobupal) (6,358)

8. TS= (Hydrochloride, Duloxetine OR Duloxetine HCl OR HCl, Duloxetine OR LY 248686 OR LY-248686 OR LY248686 OR Duloxetine Ethanedioate (1:1), (+-)-isomer - T353987 OR LY 227942 OR LY-227942 OR LY227942 OR Duloxetine OR N-methyl-3-(1-naphthalenyloxy)-3-(2-thiophene)propenamide OR N-methyl-3-(1-naphthalenyloxy)-2-thiophenepropanamine Duloxetine, (+)-isomer OR Cymbalta) (4,363)

9. (#1 AND #2) OR (#1 AND #3) OR (#1 AND #4) OR (#1 AND #5) OR (#1 AND #6) OR (#1 AND #7) OR (#1 AND #8) (355)
