# Supplementary material for: Maternal exposure to SSRIs or SNRIs and the risk of congenital abnormalities in offspring: A systematic review and meta-analysis
Source: PLoS One. 2023 Nov 29;18(11):e0294996. doi: 10.1371/journal.pone.0294996 (PMC10686472; doi:10.1371/journal.pone.0294996)
Supplement: S1 Table — (DOCX) [file pone.0294996.s002.docx]

S1 Table. Quality assessment of cohort study in the meta-analysis.

| First author (year) | Representativeness of exposed cohort | Selection of non-exposed cohort | Ascertainment of exposure | Demonstration that outcome of interest was not present at start of study | Comparability of cohorts | Assessment of outcome | Was follow up long enough for outcomes to occur | Adequacy of follow up of cohorts | Total scores |
| --- | --- | --- | --- | --- | --- | --- | --- | --- | --- |
| Ankarfeldt (2021) | * | * | * | * | ** | * | * | * | 9 |
| Kolding (2021) | * | * | * | * | ** | * | * | * | 9 |
| Huybrechts (2020) | * | * | * | * | ** | * | * | * | 9 |
| Yamamoto-Sasaki (2020) | * | * | * | * | ** | * | * | * | 9 |
| Nielsen (2017) | * | * | * | * | ** | * | * | * | 9 |
| Nishigori (2017) | * | * | * | * | ** | * | * | * | 9 |
| Petersen (2016) | * | * | * | * | ** | * | * | * | 9 |
| Jordan (2016) | * | * | * | * | * | * | * | * | 8 |
| Bérard (2015) | * | * | * | * | ** | * | * | * | 9 |
| Furu  (2015) | * | * | * | * | ** | * | * | * | 9 |
| Huybrechts (2014) | * | * | * | * | ** | * | * | * | 9 |
| Ban  (2014) | * | * | * | * | ** | * | * | * | 9 |
| Knudsen (2014) | * | * | * | * | ** | * | * | * | 9 |
| Vasilakis-Scaramozza (2013) | * | * | * | * | ** | * | * | * | 9 |
| Margulis (2013) | * | * | * | * | ** | * | * | * | 9 |
| Nordeng (2012) | * | * | * | * | ** | * | * | * | 9 |
| Malm (2011) | * | * | * | * | ** | * | * | * | 9 |
| Kornum (2010) | * | * | * | * | ** | * | * | * | 9 |
| Pedersen (2009) | * | * | * | * | ** | * | * | * | 9 |
| Merlob (2009) | * | * | * | * | - | * | * | * | 7 |
| Diav-Citrin (2008) | * | * | * | * | ** | * | * | * | 9 |

- The definition/explanation of each column of the Newcastle–Ottawa Scale is available from <http://www.ohri.ca/programs/clinical_epidemiology/oxford.asp>
- A study could be awarded a maximum of one star for each item except for the item comparability of cohorts (a maximum of 2 stars can be allotted in this item)
- Studies that controlled for age received one star, whereas studies that controlled for other factors received an additional star.
