# Supplementary material for: Maternal exposure to SSRIs or SNRIs and the risk of congenital abnormalities in offspring: A systematic review and meta-analysis
Source: PLoS One. 2023 Nov 29;18(11):e0294996. doi: 10.1371/journal.pone.0294996 (PMC10686472; doi:10.1371/journal.pone.0294996)
Supplement: S2 Table — (DOCX) [file pone.0294996.s003.docx]

S2 Table. Quality assessment of case-control studies in the meta-analysis.

| First author (year) | Is the case definition adequate? | Representativeness of the cases | Selection of controls | Definition of controls | Comparability of cases and controls | Ascertainment of exposure | Same method of ascertainment for cases and controls | Non-Response Rate | Total scores |
| --- | --- | --- | --- | --- | --- | --- | --- | --- | --- |
| Anderson (2020) | * | * | * | * | * | * | * | * | 8 |
| Werler (2018) | * | * | * | * | ** | * | * | * | 9 |
| Yazdy (2014) | * | * | * | * | * | * | * | * | 8 |
| Lind  (2013) | * | * | * | * | ** | * | * | * | 9 |
| Polen (2013) | * | * | * | * | ** | * | * | * | 9 |
| Alwan (2007) | * | * | * | * | ** | * | * | * | 9 |
| Louik (2007) | * | * | * | * | ** | * | * | * | 9 |

- The definition/explanation of each column of the Newcastle–Ottawa Scale is available from <http://www.ohri.ca/programs/clinical_epidemiology/oxford.asp>
- A study could be awarded a maximum of one star for each item except for the item comparability of cases and controls (a maximum of 2 stars can be allotted in this item)
- Studies that controlled for age received one star, whereas studies that controlled for other factors received an additional star.
