# Supplementary material for: Maternal exposure to SSRIs or SNRIs and the risk of congenital abnormalities in offspring: A systematic review and meta-analysis
Source: PLoS One. 2023 Nov 29;18(11):e0294996. doi: 10.1371/journal.pone.0294996 (PMC10686472; doi:10.1371/journal.pone.0294996)
Supplement: S4 Table — (DOCX) [file pone.0294996.s005.docx]

S4 Table. Analysis of absolute between-study variability.

|  |  | τ^2^ estimate | 95% CI |
| --- | --- | --- | --- |
| Cardiovascular system abnormalities | SSRI | 0.00 | (0.00, 0.02) |
|  | SNRI | 0.04 | (0.00, 0.20) |
| Kidney and urinary tract anomalies | SSRI | 0.01 | (0.00, 0.19) |
|  | SNRI | 0.00 | (0.00, 0.24) |
| Nervous system malformations | SSRI | 0.05 | (0.00, 0.35) |
|  | SNRI | 0.00 | (0.00, 1.60) |
| Digestive system anomalies | SSRI | 0.00 | (0.00, 0.05) |
|  | SNRI | 0.00 | (0.00, 1.30) |
| Abdominal birth defect | SSRI | 0.02 | (0.00, 0.19) |
|  | SNRI | 0.00 | (0.00, 0.27) |
| Musculoskeletal malformations | SSRI | 0.05 | (0.01, 0.13) |
|  | SNRI | 0.03 | (0.00, 0.90) |
| Eye, ear, face and neck malformations | SSRI | 0.06 | (0.00, 0.52) |
|  | SNRI | 0.00 | (0.00, 0.87) |
| Genital organs malformations | SSRI | 0.00 | (0.00, 0.72) |
|  | SNRI | 0.10 | (0.00, >100.00) |
| Respiratory system malformations | SSRI | 0.09 | (0.00, 8.80) |
|  | SNRI* | - | - |
| Overall malformation | SSRI | 0.00 | (0.00, 0.03) |
|  | SNRI | 0.02 | (0.00, 0.30) |

* There is no τ^2^ estimate because there is only one study in this subgroup.
