# Supplementary material for: Maternal exposure to SSRIs or SNRIs and the risk of congenital abnormalities in offspring: A systematic review and meta-analysis
Source: PLoS One. 2023 Nov 29;18(11):e0294996. doi: 10.1371/journal.pone.0294996 (PMC10686472; doi:10.1371/journal.pone.0294996)
Supplement: Attachment — Submitted filename: Response to Reviewers.pdf [file pone.0294996.s006.pdf]

Response letter

Dear Editor,

Thank you for your email enclosing the reviewers' comments. We have carefully reviewed the comments and have revised the manuscript accordingly. We have responded to each reviewer separately with details on how and where we have addressed the comments. For convenience, all changes to the manuscript are shown in blue.

With kind regards,

Response to Reviewer 1

1. The paper appears to have a well-defined research question and design, conducting a meta-analysis to examine the association between maternal exposure to SSRIs and SNRIs during pregnancy and congenital malformations in offspring. Meta-analyses are valuable for synthesizing existing research.

2. The paper provides a detailed methodology, including data sources, search strategies, inclusion criteria, and statistical analysis methods. This transparency is crucial for assessing the rigor of the study.

3. The paper presents a comprehensive analysis of the results, including forest plots, pooled odds ratios, and confidence intervals for various congenital malformations. The findings are well-documented and reported clearly.

4. The Discussion section is thorough, providing a thoughtful interpretation of the results, their implications, and potential clinical relevance. The authors acknowledge limitations and suggest areas for future research.

5. The paper is generally well-written, with clear language and organization. It follows a standard structure for scientific research papers.

Response: Thank you for recognizing the significance of our research and thank you so much for your positive comments.

Response to Reviewer 2

1. Overall opinion

Selective serotonin reuptake inhibitors (SSRIs) and serotonin and norepinephrine reuptake inhibitors (SNRIs) are the most common antidepressants prescribed during pregnancy, Depression is a relatively frequent disorder, and questions are raised on the risk of congenital anomalies related to these drugs. The paper intends to give an answer based on large numbers through meta-analyses of published papers. The paper is quite long, with many tables /figures, which has to be accepted by the journal, but it gives major information.

1. Recommendations

This paper will deserve publication after minor revision.

2. Detailed comments

a. Introduction

Introduction shows quite well the importance and the relevance of the raised question and is based on recent literature.

b. Material and methods

This section is well presented and is almost ok. The meta-analyses and statistical methodology is accurate. However, it would be necessary to specify in this section the categories of malformations looked for by authors. It would be also interesting to add, if possible, an analysis of the overall malformation rate. In effect, the high number of categories tested result in significant and non-significant results depending on anomalies categories and a global malformation rate would allow to better evaluate the real global risk. Moreover, it would also be interesting to give an estimate of the crude malformation percentages, which may allow the reader to see the real impact of the tested drugs on each malformation.

c. Results

Results are very well expressed and easy to read. As expressed above, it would also be interesting to give an estimate of the crude malformation risk.

d. Discussion:

This section is well written, analysing the literature, the biological hypotheses, and the strengths and weaknesses. It would be interesting to have also a discussion on the points discussed by the reviewer above.

<u>Response:</u> Thank you for your comments and suggestions. We have specified the categories of malformations in the 'Methods' section and added an analysis of the association between the overall malformation and the drugs, SSRI and SNRI in the 'Results' section. The 'Discussion' section contains comments related to this analysis.

Regarding your comment on the crude malformation percentage, we cannot calculate that percentage because our analysis included both cohort and case-control studies. Although cohort data allow prevalence calculation, case-control data do not allow prevalence estimation. However, Table 2 contains the crude prevalence of the malformations; these numbers were extracted from the CDC webpage. Hope it is acceptable to you.

Response to Reviewer 3

I had the pleasure of reviewing your manuscript, and I found your work very interesting and well-presented. However, I noticed that your analysis lacks the tau measure for assessing heterogeneity.

In your article, you only reported the I^2 measure. While this is an important measure, it is only a relative measure of heterogeneity and does not allow for assessing the prediction interval. The tau measure, on the other hand, can provide an estimate of absolute heterogeneity among the studies.

I suggest including the tau measure in your analysis. I believe that this addition could further enrich your work and provide readers with a more comprehensive understanding of the results.

Response: Thank you for your comments and suggestions. We have added the tau^2 measure to our analysis. Please see the 'Statistical Analysis' and 'Discussion' sections and the supplementary Table S4. In most cases, the value of tau^2 is equal to 0 or close to 0, indicating the studies are sufficiently similar or comparable to be pooled together.
